# Predicting hybrid rice performance using AIHIB model based on artificial intelligence

**DOI:** 10.1038/s41598-022-13805-x

**Published:** 2022-06-11

**Authors:** Hossein Sabouri, Sayed Javad Sajadi

**Affiliations:** Department of Plant Production, Collage of Agriculture Science and Natural Resources, Gonbad Kavous University, P.O. Box: 4971799151, Gonbad, Golestan Iran

**Keywords:** Plant sciences, Mathematics and computing

## Abstract

Hybrid breeding is fast becoming a key instrument in plants' crop productivity. Grain yield performance of hybrids (F1) under different parental genetic features has consequently received considerable attention in the literature. The main objective of this study was to introduce a new method, known as AI_HIB under different parental genetic features using artificial intelligence (AI) techniques. In so doing, the rice cultivars TAM, KHZ, SPD, GHB, IR28, AHM, SHP and their F_1_ hybrid were used. Having recorded Grain Yield (GY), Unfertile Panicle Number (UFP), Plant Height (HE), Days to Flowering (DF), Panicle Exertion (PE), Panicle Length (PL), Filled Grain Number (FG), Primary Branches Number (PBN), Flag Leaf Length (FLL), Flag Leaf Width (FLW), Flag Leaf Area (FLA), and Plant Biomass (BI) in the field, we include these features in our proposed model. When using the GA and PSO algorithm to select the features, grain yield had the highest frequency at the input of the Artificial Neural Network (ANN), Adaptive Neuro-Fuzzy Inference System (ANFIS) and Support Vector Machine (SVM) structure. The AI_HIB_ANN result revealed that the trained neural network with parental data enjoyed a good ability to predict the response of hybrid performance. Findings also reflected that the obtained MSE was low and R^2^ value was greater than 96%. AI_HIB_SVM and AI_HIB_ANFIS showed that measuring attributes could predict number of primary branches, plant height, days to flowering and grain yield per plant with accuracies of 99%. These findings have significant implications as it presents a new promising prediction method for hybrid rice yield based on the characteristics of the parent lines by AI. These findings contribute to provide a basis for designing a smartphone application in terms of the AI_HIB_SVM and AI_HIB_ANFIS methods to easily predict hybrid performance with a high accuracy rate.

## Introduction

Rice could be considered as a main meal for more than 60 percent of the world's population^1^. In recent years, hybrids of self-pollinating species such as wheat (*Triticum aestivum* L.), rice (*Oryza sativa* L.) and barley (*Hordeum vulgare* L.) have been considered^2^. In several nations, the production technology of hybrid rice has become prevalent. Heterosis happens when the F_1_ generation outperforms its parents' function, panicle size, grain per panicle, and branch count. According to Viermani et al., heterosis in rice differs depending on the degree of variety and alteration between parents. The *Indica* × *Japonica* crosses demonstrate the most outstanding levels of heterosis^3^. Many researchers have used hybrid and heterosis for the weight of paddy per plant and its constituent parts^4–7^.

A series of complicated features accompany the hybrid performance. The method of pollination, genomic variation, genetic basis, and adaptation play a role in this complex biological process. In addition, there are many significant variables, including inheritance of the target feature, mating method of experimental design, plant architecture, and panicle characteristics, including tiller and panicle branch^8^. Plant breeding's ultimate goal is to create high-yield variations that will boost agriculture productivity and satisfy the requirements of the developing human population. The use of hybrid breeding has shown to be an effective strategy for yield improvement. The method of hybrid rice parents is the basis of their choices. Although hybrid-breeding efforts have been a resounding achievement, selecting attractive hybrids has previously been mainly based on trial and error. Finding ideal matches between chosen parents requires a great deal of chance. One of the essential aspects of hybrid variety development is the selection of parents with the highest heterotic composition for the emergence of heterosis. The primary difficulty in hybrid breeding is predicting the success of future crosses using available information. It is costly to identify high-yielding hybrids. Predictive yield methods would help in the selection of better rice inbreed lines^9^.

The traditional method of selecting excellent hybrid combinations includes a vast number of line combinations being tested^10^. It takes a lot of time and effort to test and choose superior inbred lines for their potential to combine for hybrid production. When a significant number of inbred lines are examined, the number of hybrid combinations that may be assessed keeps rising, creating many practical challenges in performing comprehensive yield studies. As a result, the capability of correctly predicting hybrid functions based on the performance of the inbred line must be established^10^. Scientists have long been interested in estimating hybrid crop yields. For an additive and dominant genetic model, the first, best single cross predictor and choice depending on double-cross estimates are experimentally evaluated by comparing various amounts of experimental error variance and different types of hybrids^11^. There were four different techniques given by Jenkins^11^ for predicting double cross performance, three of which used single crosses, and the other one indicates the effectiveness of their use. Researchers examined projected double cross values in maize for different predictors; however, they lacked double cross data for assessing the techniques. The methods used by Eberhat^12^ were based primarily on what would be referred to as a fixed sampling plan^12^. The performance of a single cross was anticipated by utilizing the most effective linear unbiased approach depending on (i) restriction fragment length polymorphism (RFLP) data from the parental inbred and (ii) yield data from a comparable single cross set^9^. According to Bernardo (1994), parental RFLP data and relevant hybrids' yields could be used to estimate single-cross yield^9^. A link was discovered between marker polymorphisms as well as hybrid performance in rice crossings, including several germplasms^4^. Hybrid corn is predicted using the most efficient linear unbiased prediction technique^13^. He made predictions depending on current hybrids and the pedigree connection between them and untried hybrids. The efficacy of best linear unbiased prediction (BLUP) was evaluated in predicting large-scale performance, moisture, stalk, and roof lodging and offered significant evidence that BLUP may be used to identify better single crossings regularly before the field-testing process^14^. Additionally, there was also a comparison of the efficiency of the finest linear unbiased prediction based entirely on feature data (T-BLUP) and the effectiveness of the most significant linear unbiased prediction depending on feature and marker data combined (TM-BLUP), with the results indicating that the effectiveness of TM-BLUP for predicting single-cross performance as well as population breeding values^15^.

Rice hybrid performance was estimated using the most acceptable linear unbiased genomic prediction^16^. Studies have shown that imbalanced designs may benefit from mRNA transcription profiles associated with ridge-regression models even though resources are scarce and transcription profiling is restricted to a subset of genes^17^. Heterosis was evaluated utilizing a DNA marker, and it was discovered that genetic distances had a substantial impact on the degree of association due to differences in genetic inheritance as well as measurement^8^. RFLP markers were used to analyze the connection between sorghum hybrid performance and parental molecular genetic variability, with the intention of utilizing the connection to predict hybrid performance^18^.

Artificial neural networks (ANN) are now being utilized in a variety of studies. ANN with specific inputs and outputs identifies connections between every set of its inputs and their associated outputs in such applications^19^. The Multiple layer perception (MLP) is a machine learning technique that is utilized in prediction applications. MLP is mainly composed of basic perceptions organized in input, output, and one or even more hidden layers. In each layer, the number of neurons varies according to the issue condition. The supervised learning method is used to train MLPs. The training step involves feeding inputs into the network and comparing the network's outputs to the intended outputs. An error signal is produced by the variation between the actual and intended outputs. The purpose of network training is to reduce the signal of error. Error minimization is accomplished by changing network weights, with necessary calculations done by the learning algorithm. The back propagation-learning rule is utilized in the majority of instances. The weights of the layers are modified to reduce mistakes once the output layer has been computed.

According to Jang, the Fuzzy Inference System (FIS) represents uncertainty during classification and prediction issues^20^. The Takagi–Sugeno defuzzification technique was utilized by Adaptive Neuro-Fuzzy Inference System (ANFIS) to train an ANFIS network that included four stages. These stages include fuzzification of inputs, the definition of the knowledge database, rule processing, and ultimately output defuzzification (s). ANFIS's input layer forwards inputs and membership functions (MFs) to the following layer. MFs are used in the second layer for mapping input data in the range of [0, 1]. Various kinds of MFs, such as triangular, Gaussian, and bell-shaped MFs, could be used in this phase. In the rule layer, this is the third layer, each node matching the fuzzy rules’ preconditions and calculating the normalized weights. The output values arising from the inference of rules are provided by defuzzification in the fourth layer. In FIS training, two learning techniques, propagation and hybrid (a mix of propagation and least-squares approaches), are commonly utilized. The training establishes the connection between the input and output variables for determining the optimal MFs distribution. In addition, the calculation of output MF is the last change made to the ANFIS model throughout its development. There are two possible approaches: constraint-based or linear-based MF, and to get superior outcomes, both of these MFs were used. The hybrid-learning algorithm was used in this research.

Support vector machine (SVM) has excellent performance in various problems involving classification and prediction^21^. SVM showed some benefits such as quarantined performance, lower susceptibility to local minima and higher immunity to increased model complexity. Despite ANN, SVM offers excellent generalization on prediction and classification problems.

To predict hybrid performance, researchers have suggested different factors such as genomic markers^22,23^, transcriptome profiles^24–26^, metabolomic markers^16^ and phenomic markers^27^ of parental inbred lines, which are needed for performance prediction, but, in this project, hybrid performance was estimated by cross-parental characteristics with the aid of artificial intelligence (AI). The aim of this study was to estimate the hybrid yield based on parental characteristics using ANN, ANFIS and SVM models and 9 Iranian rice hybrids. To achieve this goal, we presented method AI_HIB.

## Result and discussion

### Feature selection

The phenotypic characteristics of the plant are shown by many variables that do not have the same effect or importance in predicting yield. For this reason, it is necessary to find important variables and eliminate other additional variables that may reduce the accuracy of the prediction models. Genetic Algorithm (GA) and Particle Swarm Optimization (PSO) algorithm were used to select the most important features for each cross. Selected features were used for subsequent analysis and prediction of hybrid performance. To determine which feature had the most impact on the predictions, their frequency was calculated at all crosses.

When the GA algorithm was used to select the features, Grain Yield (GY), Panicle Length (PL), Plant Height (HE) and Flag Leaf Area (FLA) with 7, 6, 5 and 5 had the highest frequency at the input of the ANN structure, respectively (Table 1). But when the PSO algorithm was used to select the features, GY, Days to Flowering (DF), Flag Leaf Length (FLL) , FLA and Plant Biomass (BI) had the highest frequency (7, 6, 6, 6 and 5, respectively).

**Table 1:** Frequency of presence in the models (feature selection).

Features	GA			PSO		
	ANN	ANFIS	SVM	ANN	ANFIS	SVM
GY	7	6	6	7	6	6
UFP	0	2	0	0	2	0
HE	5	5	5	6	5	5
DF	2	2	2	3	1	2
PE	2	2	2	2	2	2
PL	6	6	6	4	4	4
FG	3	1	3	2	2	2
PBN	2	1	1	2	1	1
FLL	3	3	3	6	5	6
FLW	2	2	3	2	2	2
FLA	5	6	6	6	6	5
BI	4	4	5	5	5	5

In the case of ANFIS model, the GA algorithm selected GY, PL, FLA and HE with frequencies of 6, 6, 6 and 5, respectively. But when the PSO algorithm was used to select the features, GY, FLA, HE, FLL and BI, respectively, had 6, 6, 5, 5 and 5, respectively, at the input of the ANFIS structure. In this attribute selection method, UFP was not inserted in the ANN structure of any of the hybrids.

In order to perform SVM analysis, when the GA algorithm was used to select the features, GY, PL, FLA, HE and BI with frequencies of 6, 6, 6, 5 and 5 had the highest presence at the input of the SVM structure, respectively. But when the PSO algorithm was used to select the features, GY, FLL, HE, FLA and BI had the highest frequencies (6, 6, 5, 5 and 5 respectively). In all modeling methods of this study and in both attribute selection algorithms, Unfertile Panicle Number (UFP), Primary Branches Number (PBN), Panicle Exertion (PE), Flag Leaf Width (FLW) and Filled Grain Number (FGN) had less frequency, respectively.

### AI_HIB_ANN: prediction of hybrid grain yield using ANN

**Figure 1:**
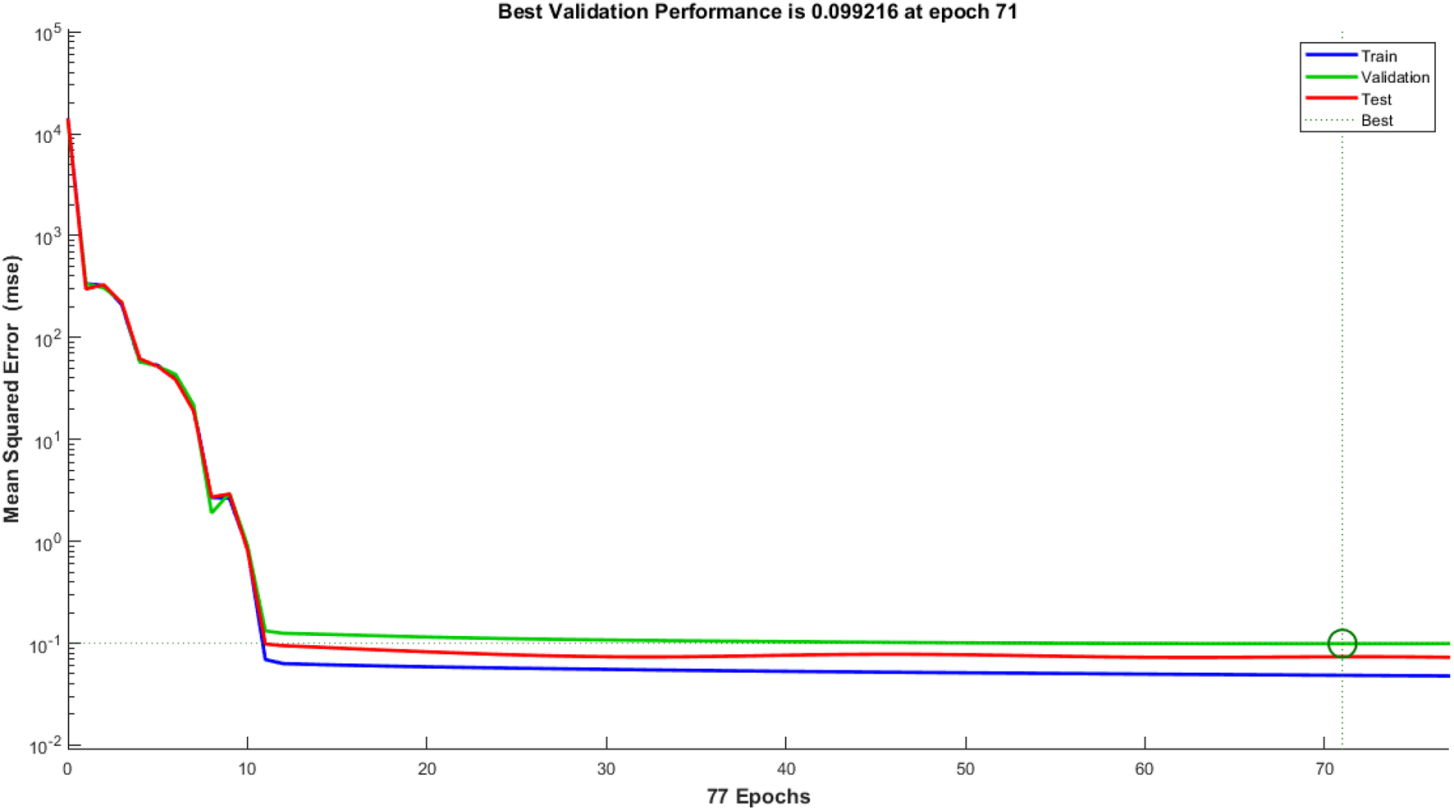
Performance of Optimal ANN in general data trained with GA selected features expressed as MSE. The MSE of selected ANN in train stage (Blue line) is lower than validation (green line) and test stages (red line) during 77 epochs. After 77 epochs ANN training stopped because of overfit avoiding as best validation MSE achieved.

**Figure 2:**
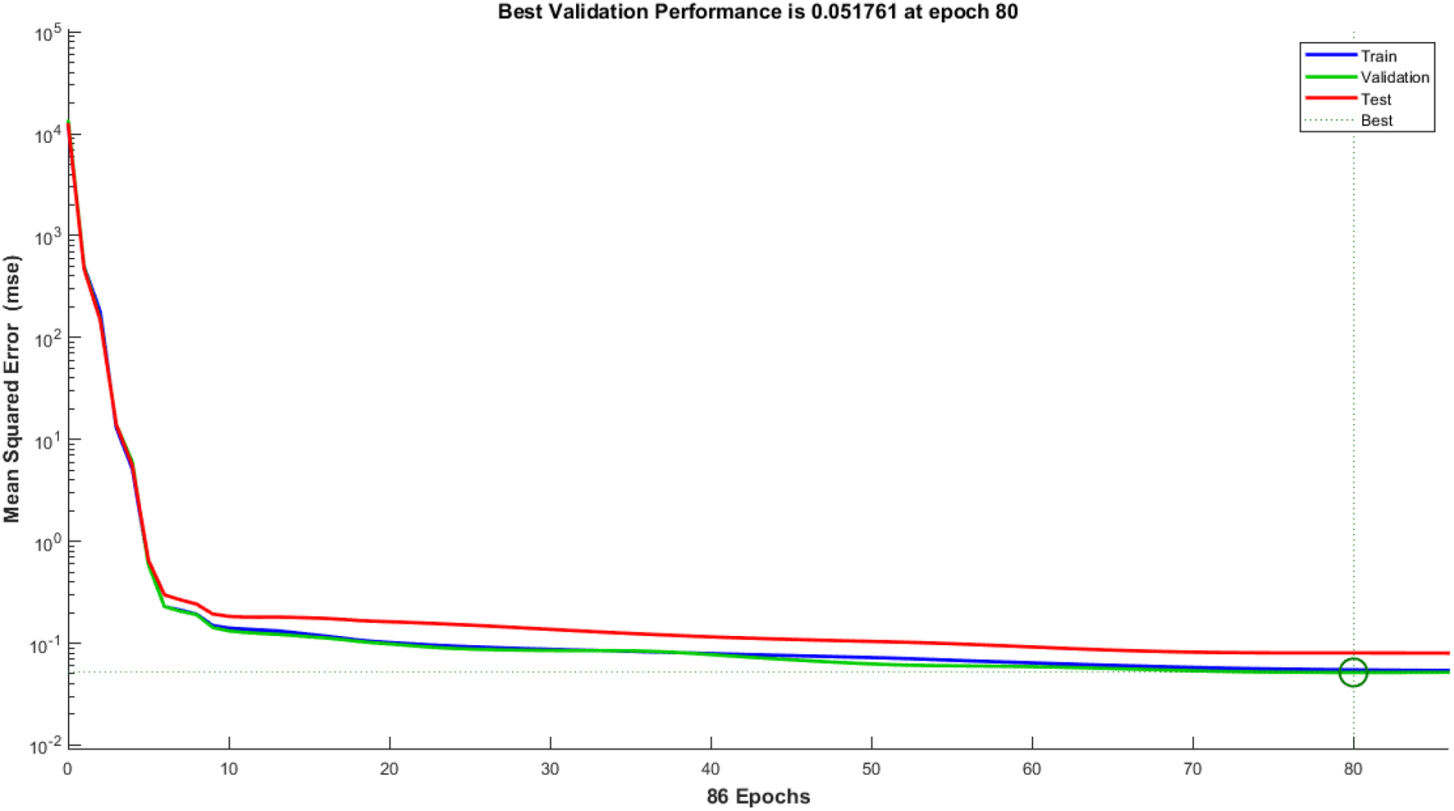
Performance of Optimal ANN in general data trained with PSO selected features expressed as MSE. The MSE of selected ANN in train stage (Blue line) is lower near validation stage (green line) and lower than test stages (red line) during 86 epochs. After 80 epochs ANN training stopped because of overfit avoiding as best validation MSE achieved.

Nine crosses were created between Taromahalli (TAM), Khazar (KHZ), Spidroud (SPD), Gharib (GHB), IR28, Ahlamitarum (AHM), and Shahpasand (SHP). The best set of ANN inputs was determine by GA and PSO algorithm. Results from train, validation and test of ANN with different structures and learning algorithms are shown briefly in Table 2. The results showed that ANN trained with four inputs contain GY, PL, FLA, and BI had the least of test MSE and belonged to TAM × SHP (Table 2). Comparison of statistical parameters of neural network performance including MSE and coefficient of determination (R^2^) in predicting of hybrid yield showed that MLP neural network with 4–34–1 structure and Levenberg–Marquardt training algorithm predicted the hybrid yield response (belong to TAM × SHP) using GA algorithm with MSE equal to 0.00076, 0.00110 and 0.00114 predicted for training, validation and test, respectively. These values were equal to 0.00094, 0.00142 and 0.00126 for PSO algorithm (with 4–31–1 structure). In order to understand that the results obtained are also true for the general data, the models were fitted to all hybrids. The results showed the power of AI in estimating hybrid performance. The MSE parameter values of this network during the training, validation and testing steps are shown in Fig. 6 (GA algorithm) and 7 (PSO algorithm). Also, as so as avoid over-fitting the network and MSE increase in validation data, network training was stopped after 77 (algorithm GA) and 80 (algorithm PSO) repetitions. Given the low value of MSE and the value of more than 96% for R^2^ in all hybrids, it can be concluded that the trained neural network with this data has a good ability to predict the response of hybrid performance. Graphs of network gradient variation including the adaptive parameter µ and the number of validation failures corresponding to each iteration during the training process are shown in Fig. 1 (GA algorithm) and Fig. 2 (PSO algorithm). At the end of the network training process, the gradient values, parameter µ and the number of validation failures are equal to 0.282, 0.001 and 6, respectively for GA algorithm. These values were 0.705, 0.001 and 6 for the PSO algorithm, respectively. The highest MSE belonged to AHM × SPD.

**Table 2:** Result of ANN in prediction of hybrid rice yield from their parent’s features.

Hybrids	FS algorithm	Selected features	ANN structure	MSE (train)	MSE (vald)	MSE (test)	R^2^ (test)
AHM × KHZ	GA	FLL, FLW, FLA, BI, FGN	5–4–1	0.0348	0.0427	0.0275	0.9684
	PSO	FLW, FLA, FGN, FLL	4–8–1	0.0250	0.0673	0.0105	0.9694
AHM × SPD	GA	PE, HE, PL, DFL, GY	5–31–1	0.0981	0.1691	0.1262	0.9695
	PSO	FLL, PL, DFL, PE, HE, GY	6–31–1	0.0755	0.6932	0.1220	0.9694
GHB × KHZ	GA	FGN, FLA, BI, PL	4–6–1	0.0069	0.0038	0.0094	0.9688
	PSO	FGN, FLL, FLA, BI	4–9–1	0.0055	0.0093	0.0106	0.9682
IR28 × GHB	GA	PE, GY, BI, HE, PL	5–37–1	0.0086	0.0117	0.0088	0.9696
	PSO	GY, PL, PE, BI, HE	5–28–1	0.0054	0.0090	0.0090	0.9696
IR28 × TAM	GA	FLA, PL, GY, HE, FLW	5–40–1	0.0011	0.0126	0.0027	0.9697
	PSO	PL, GY, HE, FLA, FLW	5–41–1	0.0011	0.0046	0.0020	0.9698
SHP × GHB	GA	GY, PBN, FLL, PL, HE	5–33–1	0.0004	0.0020	0.0016	0.9695
	PSO	GY, PBN, FLL, HE, PL	5–42–1	0.0005	0.0040	0.0012	0.9694
SHP × SPD	GA	FLL, GY, FLA, HE	4–28–1	0.0055	0.0146	0.0056	0.9697
	PSO	FLL, BI, HE, FLA, GY	5–15–1	0.0060	0.0230	0.0080	0.9696
TAM × KHZ	GA	DFL, FLW, FLA, FGN, BI	5–30–1	0.0226	0.3924	0.0399	0.9658
	PSO	FLW, BI, DFL, FLA	4–19–1	0.0221	0.07027	0.0336	0.9678
TAM × SHP	GA	FLA, PL, BI, GY	4–34–1	0.0008	0.0011	0.0011	0.9697
	PSO	FLL, BI, FLA, GY	4–31–1	0.0009	0.0014	0.0013	0.9695
General data	GA	PBN, FGN, HE, DFL, GY	5–42–1	0.0486	0.0992	0.0738	0.9699
	PSO	PBN, GY, DFL, FGN, HE	5–32–1	0.0550	0.0518	0.0805	0.9698

**Figure 3:**
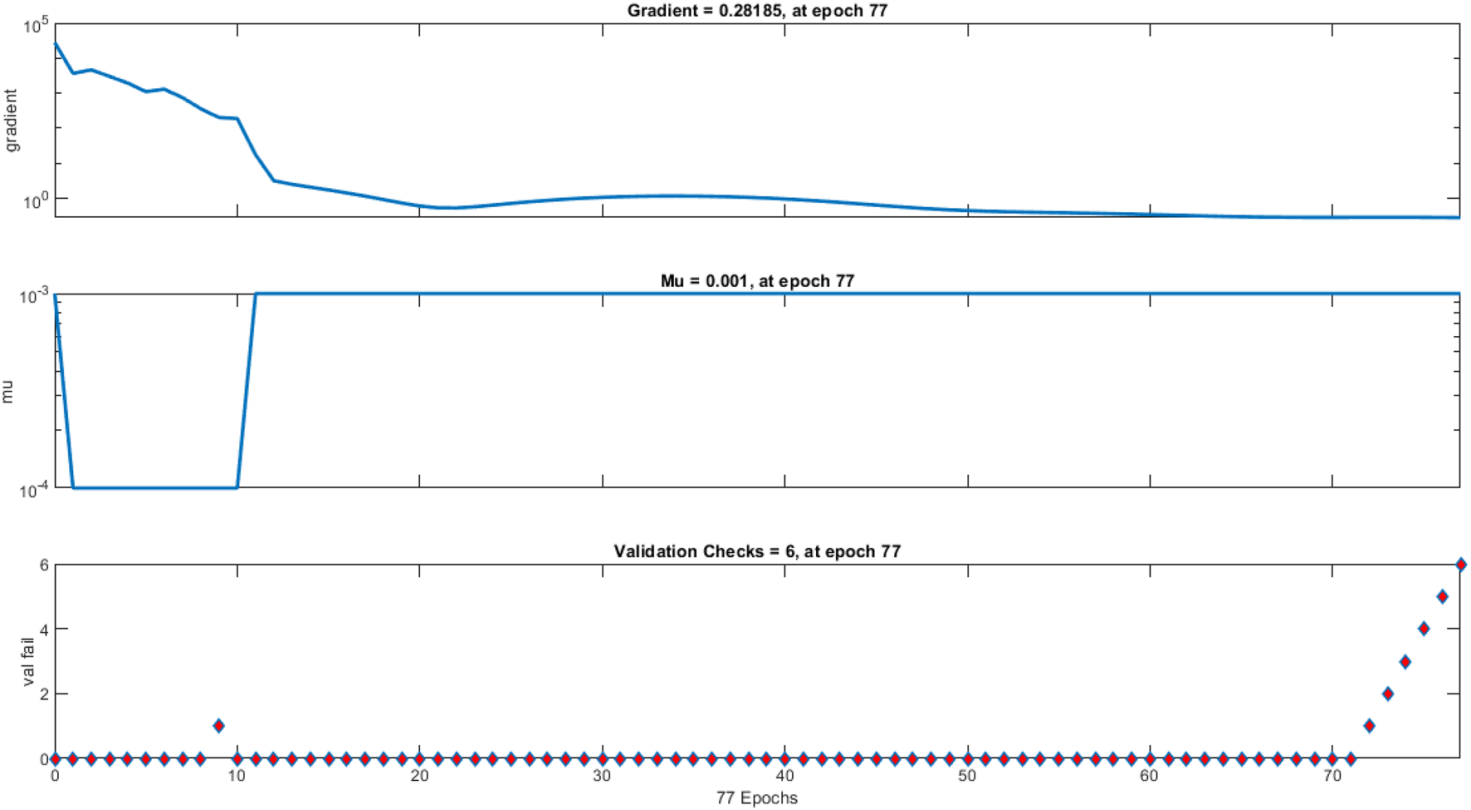
Controlling values of how neural network training, A. Gradient values in the network training phase for each iteration, B. µ values for each iteration, and C. validation values for each iteration using GA feature selection algorithm.

**Figure 4:**
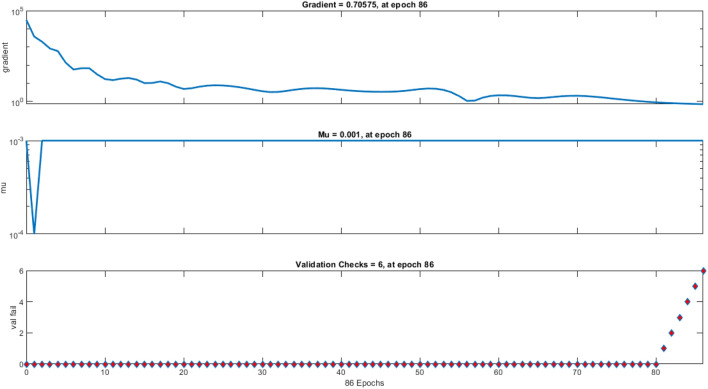
Controlling values of how neural network training, (**A**) Gradient values in the network training phase for each iteration, (**B**) µ values for each iteration, and (**C**) validation values for each iteration using PSO feature selection algorithm.

Hybrid grain yield predicted was fitted on actual hybrid grain yield with regression and goodness of fitness test. Despite difference between the MSE test values ​​obtained for the hybrids, for all of them the R^2^ value between the predicted and actual values ​​was estimated above 96% that showed in Fig. 3 (GA algorithm) and Fig. 4 (PSO algorithm). The goodness of fitness test did not reveal a differences between actual and estimated data. Although feature selection algorithms for each hybrid introduced specific attributes into the prediction model, but the results showed that estimates can be made with robust and high accuracy for all type of hybrids.

Hybrid grain yield prediction has attracted a huge deal of interest. According to Westhues and Schrag (2017), integration of genomic and transcriptomic data is effective in the prediction of hybrid maize’s important agronomic properties^28^. Wang et al. compared the predictabilities from all integrations of three omic data utilizing eight conventional prediction approaches^29^. They concluded that integrating the metabolomic and genomic data normally presents the best prediction in rice. The hybrid prediction in terms of metabolomics and genomic data has been progressed. However, it is still a challenge to maximize the predictability as accurate, easy, and accessible to all. Mainly, former omic predictions for hybrid performance were concentrated on transcriptomic, genomic, and metabolomic data. However, the phenotypic information of parents (phenome) was overlooked. Indeed, phenotypes are the core of crop breeding. Moreover, experienced breeders can guess the performance of hybrids considering the phenotypes of their parents, to some degree^30^. Several studies have been performed on the prediction of hybrid yield^31,32^, though it is still not clear whether integration of AI approaches can enhance the hybrid prediction.

**Figure 5:**
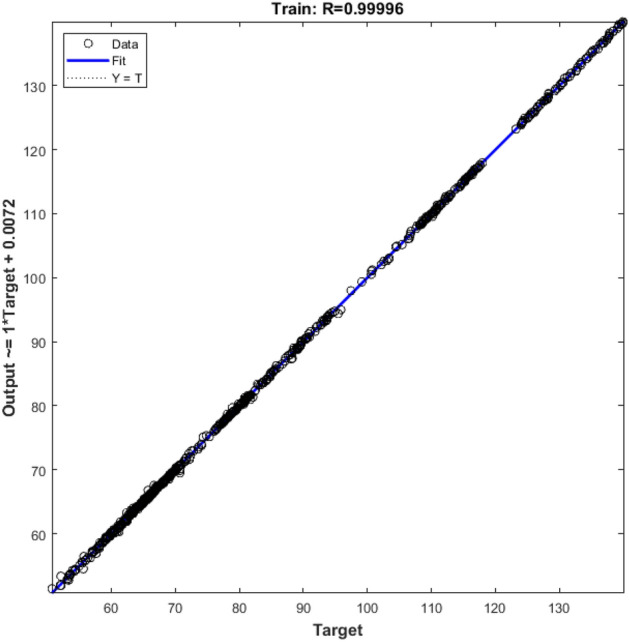
Linear regression between actual values and predicted by the neural network in the train stage using GA feature selection algorithm. Data points are shown as small circles and regression line fitted on these data points.

**Figure 6:**
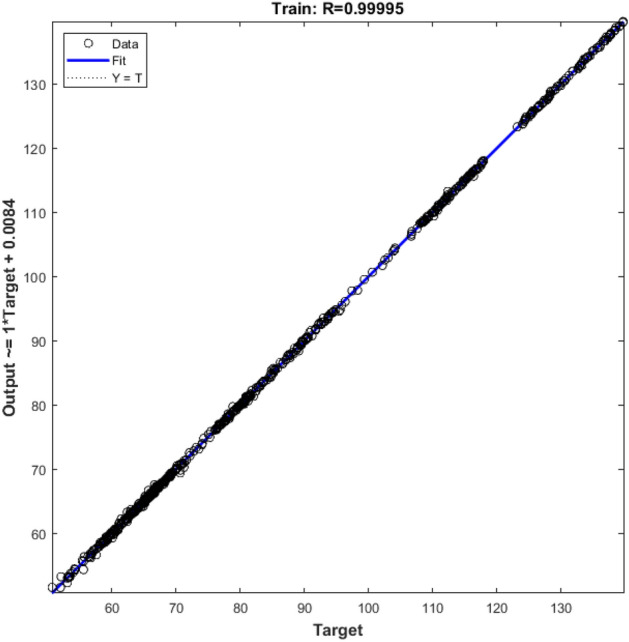
Linear regression between actual values and predicted by the neural network in the train stage using PSO feature selection algorithm. Data points are shown as small circles and regression line fitted on these data points.

According to these result, neural network methods enable to predict the performance of the hybrid using parent’s characters. Linear regression between actual values and predicted by the neural network in the test stage using GA and PSO feature selection algorithm presented in Figs. 5 and 6.

ANN can explore the nonlinear association in the input data set. Using certain learning algorithms and with the appropriate topology and correct weights of connections between neurons, neural networks can be trained for approximating each function representing the dependence of the outputs on the inputs^33^. Neural networks have some benefits, such as less formal statistical training, the capability at implicitly detecting the complex nonlinear relations between independent and dependent variables, the ability to discover all probable interactions between predictor variables, as well as the availability of multiple training algorithms^34^.

### AI_HIB_ SVM: prediction of hybrid grain yield using SVM

In the SVM method, nine groups of inputs were defined. Evaluation of statistics based on SVM model showed that the highest estimate of hybrid performance belonged to cross SHP × GHB. The GA and PSO selected features GY, HE, PL, NPB and LFL for this cross. The final model converged with the minimum mean squares error value of 0.0065 based on the GA algorithm (Table 3). At the point of convergence, the values of Box Construct, Kernel Scale and Epsilon were 0.0011, 0.3020, and 0.0090, respectively. Also, based on the PSO algorithm, the final model converged with a minimum mean squares error value of 0.0059. At the convergence point, the values of Box Construct, Kernel Scale and Epsilon were 0.0011, 0.302, and 0.0090, respectively. The determination of coefficients for them were 99.76 and 99.77, respectively. Evaluation of other hybrids and general data showed that the highest mean squares error and the lowest determination of coefficient belonged to hybrid TAM × KHZ. The estimated performance of hybrids for general data was also higher than 90%. No significant differences showed between the predicted and observed hybrid grain yield for all hybrid and general data. The results showed that by measuring attributes grain yield, days to flowering, plant height filled grain number and Primary branches number, the yield of hybrids can be predicted with 93% higher accuracy.

**Table 3:** Result of SVM in prediction of hybrid rice yield from their parent’s features.

Hybrids	FS algorithm	Selected features	Box construct	Kernel scale	Epsilon	MSE (train)	MSE (test)	R^2^ (test)
AHM × KHZ	GA	FLL, FLW, FLA, BI, FGN	0.0010	0.0037	0.0136	0.3906	0.3832	0.9825
	PSO	FLW, FLA, FGN, FLL	0.07909	0.0251	0.4079	0.4208	0.3139	0.9856
AHM × SPD	GA	PE, HE, PL, DFL, GY	0.4446	0.1168	0.9978	0.6617	0.8357	0.9971
	PSO	FLL, PL, DFL, PE, HE, GY	0.0089	0.0198	0.6951	0.6587	0.7756	0.9972
GHB × KHZ	GA	FGN, FLA, BI, PL	0.0010	1.1356	0.1000	0.2063	0.0896	0.9881
	PSO	FGN, FLL, FLA, BI	0.3554	0.1798	0.5457	0.1633	0.1333	0.9865
IR28 × GHB	GA	PE, GY, BI, HE, PL	0.0250	0.1249	0.0200	0.0854	0.0893	0.9963
	PSO	GY, PL, PE, BI, HE	13.7706	2.2529	0.0199	0.0858	0.0916	0.9963
IR28 × TAM	GA	FLA, PL, GY, HE, FLW	0.1610	0.4647	0.1681	0.0426	0.0320	0.9965
	PSO	PL, GY, HE, FLA, FLW	0.1178	0.1279	0.1599	0.0242	0.0227	0.9975
SHP × GHB	GA	GY, PBN, FLL, PL, HE	0.0011	0.0302	0.0090	0.0049	0.0065	0.9976
	PSO	GY, PBN, FLL, HE, PL	0.0012	0.0011	0.0022	0.0043	0.0059	0.9977
SHP × SPD	GA	FLL, GY, FLA, HE	228.3724	2.2274	0.0226	0.8484	0.1181	0.9947
	PSO	FLL, BI, HE, FLA, GY	0.1388	0.0296	0.0573	0.9075	0.1222	0.9945
TAM × KHZ	GA	DFL, FLW, FLA, FGN, BI	1.0898	10.8553	1.3632	1.5613	1.3631	0.9177
	PSO	FLW, BI, DFL, FLA	836.1961	94.8378	2.2235	1.4607	1.2604	0.9225
TAM × SHP	GA	FLA, PL, BI, GY	0.0010	0.4030	0.0440	0.0392	0.0140	0.9956
	PSO	FLL, BI, FLA, GY	69.4411	0.0010	0.3828	0.0282	0.0260	0.9956
General data	GA	PBN, FGN, HE, DFL, GY	884.6082	9.2008	4.6115	41.650	40.278	0.9303
	PSO	PBN, GY, DFL, FGN, HE	184.7721	9.8048	4.9218	42.030	40.641	0.9296

SVMs have been used successfully in various research areas. These systems are oriented by the structural risk minimization, rather than the empirical risk minimization of the ANN. Using the empirical risk minimization causes the overfitting problem for the network since the solution is captured at a local minimum. The model complexity and empirical error are simultaneously minimized by structural risk minimization. Then, the SVM’s generalization ability for regression problems or classification can be enhanced in several disciplines^35^. SVM is a very convenient method for predicting dependent variables in various sciences. For example, the following can be mentioned: wall Parameters in Through-Wall Radar Imaging^36^, Wafer Yield^37^, Aqueous Solubility^38^, and drag coefficient^39^, Conceptual Cost Estimation in Construction Projects^40^, Evapotranspiration^41^, iron concentration^42^ and total Organic Carbon^43^. Thus, based on the SVM method, to predict hybrid performance, we need four attributes to achieve the best results to estimate the hybrid performance using parents’ features.

### AI_HIB_ ANFIS: prediction of hybrid grain yield using ANFIS

**Figure 7:**
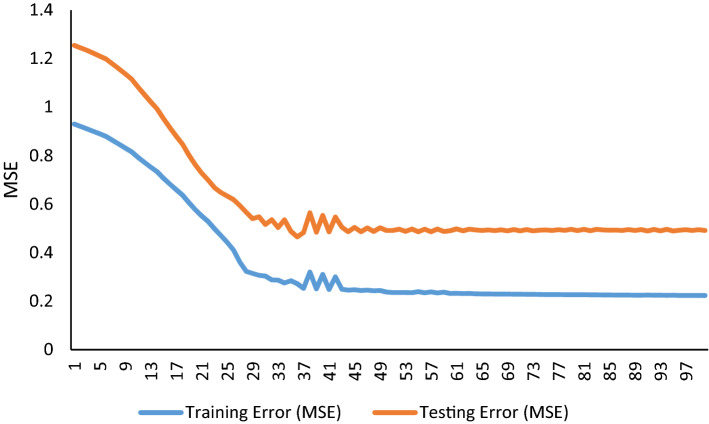
ANFIS performance trained with GA selected features. Training error (blue line) is lower than testing error (red line) expressed as MSE over training epochs.

**Figure 8:**
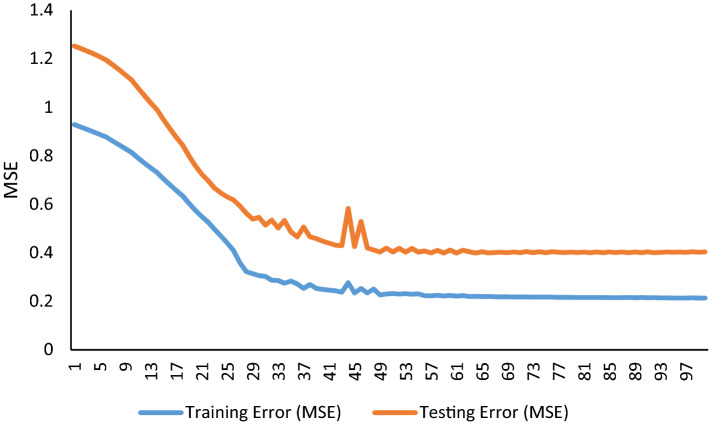
ANFIS performance trained with PSO selected features. Training error (blue line) is lower than testing error (red line) expressed as MSE over training epochs.

**Figure 9:**
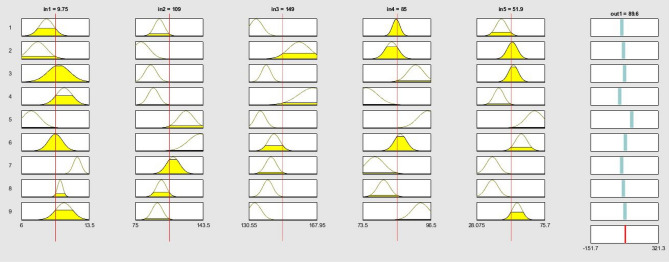
Fuzzy rules used in ANFIS training using GA feature selection algorithm. The ANFIS structure consists of 5 inputs and one output and 9 rules. The last row in output column represents the final calculated ANFIS output.

**Figure 10:**
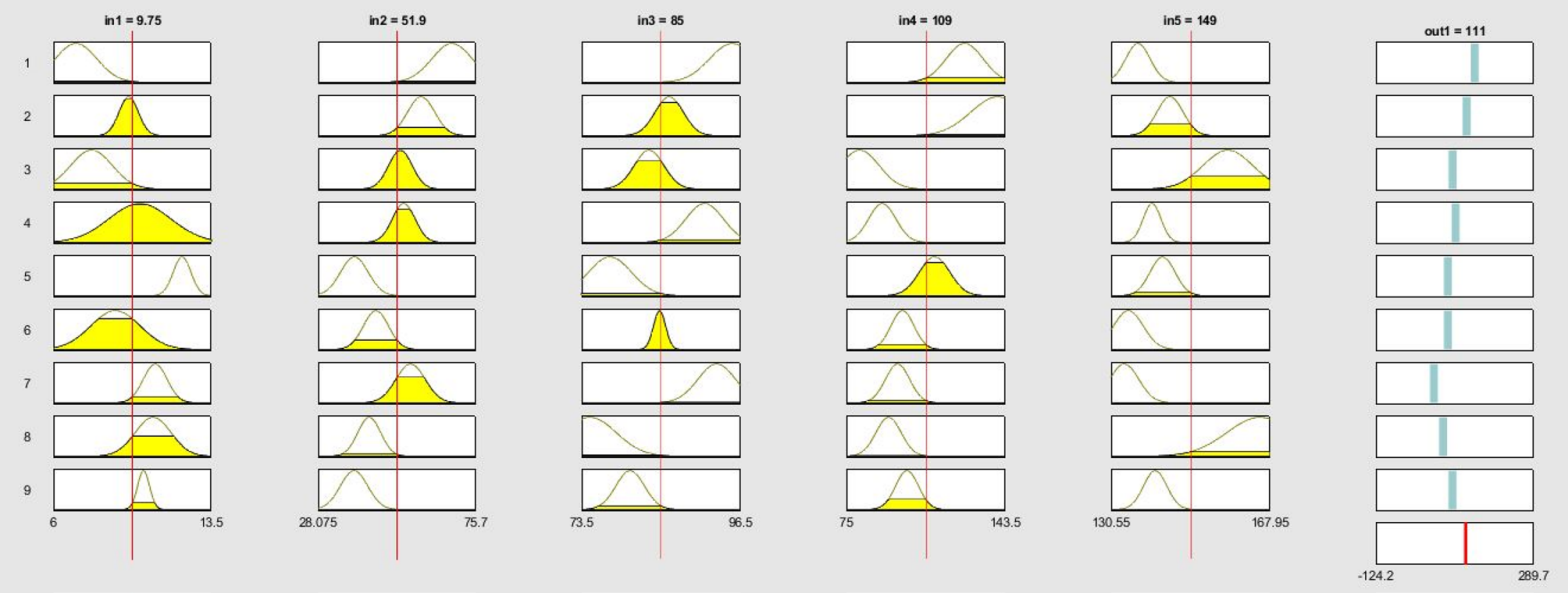
Fuzzy rules used in ANFIS training using PSO feature selection algorithm. The ANFIS structure consists of 5 inputs and one output and 9 rules. The last row in output column represents the final calculated ANFIS output.

Nine groups of inputs (belong to different hybrids) were defined in ANFIS. The highest estimate of hybrid performance belonged to cross SHP × GHB. The GY, HE, PL, NPB and LFL selected using GA and PSO algorithms for this cross. The final model converged with the test mean squares error value of 0.002621 and 0.002663 on the base of GA and PSO algorithm, respectively (Table 4). Also, final model converged with a train mean squares error value of 0.000894 and 0.000888 on the base of GA and PSO algorithm, respectively. Accuracy of estimates in this cross were very close to TAM × SHP cross. The determination of coefficients for them were 99.90. Evaluation of other hybrids and general data showed that the highest mean squares error and the lowest determination of coefficient belonged to hybrid TAM × KHZ. The estimated performance of hybrids for general data was also higher than 99%. No significant differences showed between the predicted and observed hybrid grain yield for all hybrid and general data. The results showed that by measuring attributes number of primary branches, umber of filled grain, height, days to flowering and grain yield per plant, the yield of hybrids can be predicted with 99% higher accuracy. The same Features proposed based on the SVM model. ANFIS performance in training and testing using GA and PSO feature selection algorithm and Fuzzy rules used in ANFIS training using GA feature selection algorithm presented in Figs. 12, 13, 14 and 15. Also, The ANFIS structure consists of 5 inputs and one output and 9 rules using GA and PSO feature selection algorithm, Input membership functions 1–4 using GA and PSO feature selection algorithm and step size chart for trained ANFIS network using GA feature selection algorithm presented Figs. 7, 8, 9, and 10.

**Table 4:** Result of ANFIS in prediction of hybrid rice yield from their parent’s features.

Hybrids	FS algorithm	Selected features	MSE (train)	MSE (test)	R^2^ (test)
AHM × KHZ	GA	FLL, FLW, FLA, BI, FGN	0.0031	0.1335	0.9937
	PSO	FLW, FLA, FGN, FLL	0.0123	0.0927	0.9961
AHM × SPD	GA	PE, HE, PL, DFL, GY	0.1096	0.1791	0.9993
	PSO	FLL, PL, DFL, PE, HE, GY	0.0905	0.2925	0.9989
GHB × KHZ	GA	FGN, FLA, BI, PL	0.0110	0.0196	0.9974
	PSO	FGN, FLL, FLA, BI	0.0102	0.0284	0.9962
IR28 × GHB	GA	PE, GY, BI, HE, PL	0.0089	0.0396	0.9985
	PSO	GY, PL, PE, BI, HE	0.0089	0.0396	0.9984
IR28 × TAM	GA	FLA, PL, GY, HE, FLW	0.0034	0.0050	0.9994
	PSO	PL, GY, HE, FLA, FLW	0.0034	0.0050	0.9994
SHP × GHB	GA	GY, PBN, FLL, PL, HE	0.0009	0.0026	0.9990
	PSO	GY, PBN, FLL, HE, PL	0.0009	0.0027	0.9990
SHP × SPD	GA	FLL, GY, FLA, HE	0.0078	2.1419	0.9245
	PSO	FLL, BI, HE, FLA, GY	0.0073	2.0752	0.9257
TAM × KHZ	GA	DFL, FLW, FLA, FGN, BI	0.0617	0.3850	0.9821
	PSO	FLW, BI, DFL, FLA	0.0530	0.3332	0.9865
TAM × SHP	GA	FLA, PL, BI, GY	0.0009	0.0028	0.9991
	PSO	FLL, BI, FLA, GY	0.0011	0.0027	0.9991
General data	GA	PBN, FGN, HE, DFL, GY	0.2725	0.4651	0.9992
	PSO	PBN, GY, DFL, FGN, HE	0.2219	0.3993	0.9993

ANFIS as an adaptive network allows the use of neural network topology along with fuzzy logic. It comprises the features of both methods and removes some disadvantages of using them alone. ANFIS operation is similar to the feed-forward backpropagation network. Calculation of the consequent parameters is forward. However, premise parameters are determined backward. The neural section of the system includes two learning methods of the hybrid learning method and the back-propagation learning method. Only zero or first-order Sugeno inference system or Tsukamoto inference system can be utilized in the fuzzy section^44,45^.

The ANFIS technique has been widely used in various sciences as follow: number of foreign visitors^46^, Outdoor Temperaturoft Sensors^47^, acid solvent solubility in supercritical CO_2_^48^, solar radiation^49^, Roadheader Performance from Schmidt Hammer Rebound Values^50^, degree of polymerization using dissolve gas analysis and oil characteristics ^51^, PCUs at Different Levels of Service^52^, housing demand^53^ and evapotranspiration^54^. Although the ANFIS method has been used in various sciences to predict dependent variables, this study is the first report of predicting hybrid performance using this technique.

The methods proposed in this study can be used by breeders to predict hybrid seed yield. This type of machine learning method is useful for decision makers in two ways. First, because only the phenotypic characteristics of inbred lines are used to develop the model, it helps breeders reduce costs by reducing the number of hybrid breeding trials. Second, the breeding process will no longer be time consuming because there is no need to wait for the results of field experiments. This information can be obtained very quickly from the model.

## Conclusion

Hybrid grain production technology is one of the most important parts of plant breeding. The biggest challenge in hybrid breeding is how to predict the performance of future crosses based on existing Hybrid. The prediction of the hybrid action has long been the subject of research by plant breeders. Identifying high yielding hybrids is expensive. Methods for predicting hybrid yield would facilitate the identification of superior rice inbreed lines^9^.

Unlike conventional rice breeding (inbreeding following two-way or three-way cross-breeding and release), hybrid rice breeding is proposed to increase grain yield by exploiting the heterosis phenomenon. Extensive field evaluations are required to estimate hybrid rice yield. This makes predicting hybrid rice based on parental line phenotype an important strategy. Phenotyping a wide range of hybrids is a fundamental step in predicting unobserved hybrids. To avoid the high cost of testing produced hybrids, the use of AI is the best strategy.

Conventional selection of superior hybrid combinations involves testing of large numbers of line combinations^8^. Testing and selection of superior inbred lines for their combining ability for hybrid production demands a great amount of effort. When a high number of inbred lines are tested, the possible number of hybrid combinations to be evaluated in tremendously high. This poses a lot of practical difficulties in conducting extensive yield tests. Therefore, with the ability to accurately predict the performance of hybrids from the performance of inbred lines need to be developed^10^.

To compare the three prediction methods, the coefficient of determination were compared. The values of t for comparison of AI_HIB_ANN and AI_HIB_SVM, AI_HIB_ANN and AI_HIB_ANFIS, finally AI_HIB_SVM and AI_HIB_ANFIS were − 0.01082, − 0.02038 and − 0.00957, respectively. The P-value values between the above comparisons were 0.102, 0.001 and 0.263, respectively. Due to the higher mean of AI_HIB_SVM (98.00%) and AI_HIB_ANFIS (98.95%) compared to AI_HIB_ANN (96.92%) method, we recommend the AI_HIB_SVM and AI_HIB_ANFIS method to predict hybrid performance.

The ANN method needs a lot of data for training and learning. Moreover, the correlation between the inputs and output is very crucial for better performance of the ANN. In addition, the weight and bias of the hidden layer and output layer need to be properly tuned during the training period to get better performance^55^.

On the other hand, the adaptive ANFIS is a hybrid system with the benefits of both ANN and the fuzzy system. Therefore, the ANFIS performs better than the ANN for prediction. ANFIS has the capability of fast learning, effective handling of uncertainty and imprecision^56^.

The SVM method is accurate and it is capable of minimizing the over-fitting issue. SVM can provide prediction result based on limited set of information, it is useful when the parameters are optimized by other intelligent methods. Compared with the other traditional machine learning, SVM possesses stronger generalization performance. When used for regression forecasting, SVM has the advantages of avoiding falling into local optimum compared to other nonlinear prediction models. SVM is a viable alternative to ANN in hybrid yield prediction due to its stability and good performance. SVM shows the strong resistance to the over-fitting problem and the high generalization performance. It is mainly because SVM can construct a mapping from one-dimensional input vector into high-dimensional space by the use of reproducing kernels^57^.

Refer to Table 4 to determine which parental Features to measure the performance of hybrids. As it is known, GY, HE, PL and FLA attributes have the highest presence rate in feature selection algorithms. Therefore, it is suggested that the above features be measured in parents.

In this study, for the first time, hybrid performance was estimated by cross-parental characteristics with the help of AI. In all the methods that have been proposed so far to estimate the performance of the hybrid, we require laboratory costs and the employment of specialized personnel. But in the AI-based methods discussed in this study, we only need to measure to some of parent features. With the help of AI, it is easy to select from a large number of possible cases for crossing between inbred lines, a limited number for its done. This achievement can contribute significantly to the success of rice breeders.

The models obtained in this research can be used in the different environmental conditions. We tried to minimize this effect by increasing the number of crosses in one environment. More research is needed to develop global models and make them usable in various environments. Therefore, it is suggested that these experiments be repeated in different locations with different environmental conditions. We recommended that the model presented in this article be used for other environments to test its globality.

## Material and methods

### Field considerations

#### Location of field experiment

Experiments were performed at Gonbad Kavous University. The location of the experimental field is at 17° 37 latitudes, 12° 55 longitudes, and 45 m above sea level. The soil in the performed experimental plots was Si.Cl.L in texture. Some of physical and chemical properties of soil presented in Table 5.The experiments were conducted during June 2017 and 2018.

**Table 5:** Some of physical and chemical properties of soil.

Characteristics	Content
Electric conductivity (dS m^−1^)	2
pH	7.6
Neutralizing agents (percent)	10.5
Organic carbon (percent)	1.6
Total nitrogen (percent)	0.11
Absorbable phosphorus (ppm)	12.3
Absorbable potassium (ppm)	414
Silt (percent)	55
Clay (percent)	32
Sand (percent)	13
Soil texture	Si.Cl.L
Fe	4
Mn	8.17
Zn	0.7
Cu	2

#### Crossing the genotypes and agricultural operations

Nine cultivars of rice were selected comprising TAM, SPD, KHZ, GHB, IR28, AHM, and SHP. All cultivars were grown in isolated conditions completely and were totally pure. They were categorized in the *Indica* group. However, they were significantly different in terms of Mn, Fe, Zn, and protein content^58^, blast disease^59^, drought tolerance^60^, and agronomic properties^61,62^. TAM, AHM, GHB, and SHP are Iranian traditional cultivars with higher plant height, lower tiller number, low and medium yield, lodging susceptibility, low biomass, low tiller number, and lower amylose content based on quality. However, SPD, KHZ, and IR28 are improved cultivars. SPD cultivar is an enhanced cultivar developed by Damsiah/IR8 cross at Rice Research Institute of Iran (RRII). Furthermore, the KHZ as an improved cultivar was developed by TNAU7456/IR36 cross at Rice Research Institute of Iran. IR28 led to a biparental cross as lR833-6-2-1-1///lR1561-149-1//lR24*4/O nivara at International Rice Research Institute. SPD, KHZ, and IR28 have significant differences with landrace cultivars based on morphological properties, abiotic and biotic stress, as well as quality features (Tables 6, 7). Hence, landrace and improved cultivars were crossed. The crosses were performed in such a way that the parents were highly different agronomic and quality properties making the hybrid superior. The population was developed to present the plant genetic materials under the Gonbad Kavous University’s license. All the methods were performed in accordance with relevant guidelines and regulations.

**Table 6:** Parent’s attributes.

Features	TAM	KHZ	SPD	GHB	IR28	AHM	SHP
GY (gr)	34.17	41.01	58.91	51.07	85.00	34.98	53.97
UFP (no.)	12.51	11.54	20.93	23.06	15.50	18.36	14.40
HE (cm)	167.20	117.30	105.60	151.30	122.30	166.80	164.90
DFL (day)	67.90	84.54	105.15	85.25	104.96	76.63	80.91
PE (cm)	16.50	8.60	7.00	10.60	6.70	8.60	12.50
PL (cm)	33.50	30.90	19.00	30.00	24.00	26.50	31.50
FGN (no.)	92.40	132.70	103.30	67.50	188.30	73.00	92.90
PBN (no.)	11.00	12.90	10.50	4.50	8.10	9.90	10.90
FLL (cm)	23.00	27.30	20.20	36.50	24.10	23.90	30.50
FLW (cm)	1.00	1.30	1.20	1.40	1.10	1.20	1.40
FLA (cm^2^)	84.16	26.45	18.96	37.14	20.72	21.64	31.98
BI (gr)	120.89	106.96	133.71	115.65	177.57	123.10	149.37

**Table 7:** Hybrid’s attributes.

Features	AHM × KHZ	AHM × SPD	GHB × KHZ	IR28 × GHB	IR28 × TAM	SHP × GHB	SHP × SPD	TAM × KHZ	TAM × SHP
GY (gr)	60.56	87.64	62.75	131.27	113.12	78.98	87.45	65.82	67.79
UFP (no.)	18.53	30.98	30.14	29.34	23.14	31.84	25.22	16.86	18.40
HE (cm)	168.30	164.70	150.70	142.90	143.20	164.50	138.00	161.50	149.40
DFL (day)	88.44	101.24	106.95	122.63	114.04	84.96	130.58	82.58	76.41
PE (cm)	9.60	9.70	0.90	12.80	12.40	5.00	8.90	18.40	11.60
PL (cm)	32.10	33.10	35.10	33.90	33.20	32.70	33.90	35.60	34.40
FGN (no.)	179.30	132.90	165.60	228.10	224.40	90.80	130.45	145.40	96.50
PBN (no.)	14.60	12.30	14.50	11.60	10.50	10.50	13.49	15.50	11.10
FLL (cm)	29.50	34.50	31.20	35.50	25.20	35.70	28.80	32.10	29.50
FLW (cm)	1.30	1.20	1.40	1.20	1.10	1.00	1.40	1.30	1.40
FLA (cm^2^)	29.04	31.39	31.99	31.99	21.65	28.21	30.45	33.07	30.24
BI (gr)	129.67	281.79	131.10	203.48	208.84	171.45	199.63	132.97	145.55

The present work was performed on 9 crosses. KHZ and TAM were crossed in the first cross. Planting 150 seeds TAM (male parent) and 150 seeds KHZ (female parent) was performed as single seedlings. One plant of KHZ was planted near TAM. Emasculating 1/2 of the KHZ main panicle, they were pollinated by the TAM. The other half of the paternal and maternal main panicle were selfed. The seeds of the selfed and the first generation of their crosses were planted in the second year, in rows of 1-m as a single seedling. Ultimately, 5 plants were selected from each row to determine the features. The operation was performed for other crosses (AHM × SPD, GHB × KHZ, IR28 × GHB, IR28 × TAM, SHP × GHB, SHP × SPD, TAM × KHZ, and TAM × SHP).

Transplanting distances as 25 × 25 cm were used. 30 days old seedlings were transplanted in each hill with one plant per hill. After transplanting, 3-inch water depth was kept till seven days before harvest. Fertilizers were applied three times for a total amount of 200 kg/ha. The first application consisted of 25% urea at the time of field preparation, the second application consisted of 50% urea 40 days after transplanting and the last application consisted of 25% urea applied before flowering stage. Insects, diseases, and weeds were thoroughly controlled until harvesting.

#### Features recording

In all the trials GY, UFP, HE, DF, PE, PL, FG, PBN, FLL, FLW, FLA, and BI were recorded per the standard evaluation system^63^. Evaluation method and growth stage of recording presented in Table 8.

**Table 8:** Features, measurement method and growth stage of recording.

Features	Measurement method	Growth stage
GY (gr)	Weight of filled grain per plant	9 (mature grain)
UFP (No.)	Number of panicles less than 75% fertile spikelets	9 (mature grain)
HE (cm)	Measurement from soil surface to tip of the tallest panicle (awns excluded)	9 (mature grain)
DFL (day)	Number of days from planting to flowering of half of the main panicle	6 (heading)
PE (cm)	Distance between the flag leaf to the first node below the main panicle	9 (mature grain)
PL (cm)	Measurements in centimeters from panicle base to tip	8 (dough stage)
FGN (no.)	Panicles over than 75% fertile spikelets	9 (mature grain)
PBN (no.)	Number of primary branches in main panicle	9 (mature grain)
FLL (cm)	Measurements of the flag leaf	8 (dough stage)
FLW (cm)	Measurements of the widest portion of the flag leaf blade	8 (dough stage)
FLA (cm^2^)	Length multiplied by width multiplied by 0.75	8 (dough stage)
BI (gr)	Weight of plant	9 (mature grain)

### Mathematical considerations

#### Data processing

The data is divided into two sections. The input of AI models is average of parent’s attributes. Hybrid grain yield is used as the target of AI models. The Data were randomized and normalized to improve the AI models performance. All analysis was performed at MATLAB programming environment (https://www.mathworks.com) and its built-in functions from Machine Learning and Deep Learning Toolbox (Matlab Machine Learning and Deep Learning Toolbox,The MathWorks, Natick, MA, USA (2020)) and Fuzzy Logic Toolbox (Matlab Fuzzy Logic Toolbox,The MathWorks, Natick, MA, USA (2020)).

#### Feature selection

**Figure 11:**
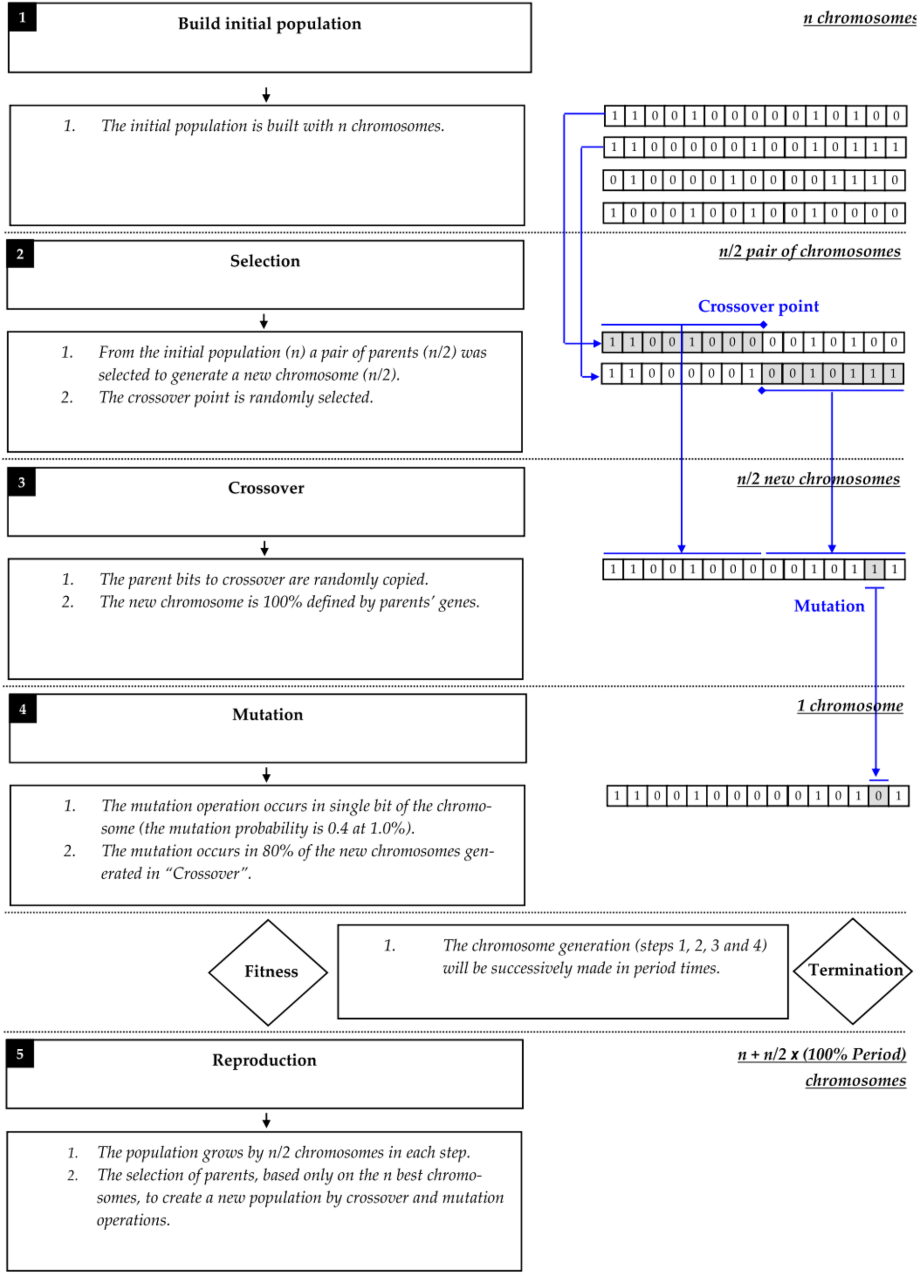
Genetic Algorithm^65^. This flowchart represents 5 main steps in GA algorithm: Build initial population, Selection, Crossover, Mutation, Reproduction. GA first starts the optimization process by generating the initial population from a random solution to the problem. Crossover components combine solutions during optimization and Mutation changes some of the solutions significantly and emphasizes the general study of search space.

The GA and PSO Algorithm were used to determine the most effective features affecting the performance of hybrids. GA is one of the population-based evolutionary algorithms that uses the simulated model population search to find the optimal values of parameters. GA first starts the optimization process by generating the initial population from a random solution to the problem. The initial random population is repeatedly evaluated by the fitness function and evolved to minimize or maximize goals. The main operators in GA are crossover and mutation. Crossover components combine solutions during optimization and are the main tools for exploring search space. Mutation changes some of the solutions significantly and emphasizes the general study of search space (Fig. 11).

**Figure 12:**
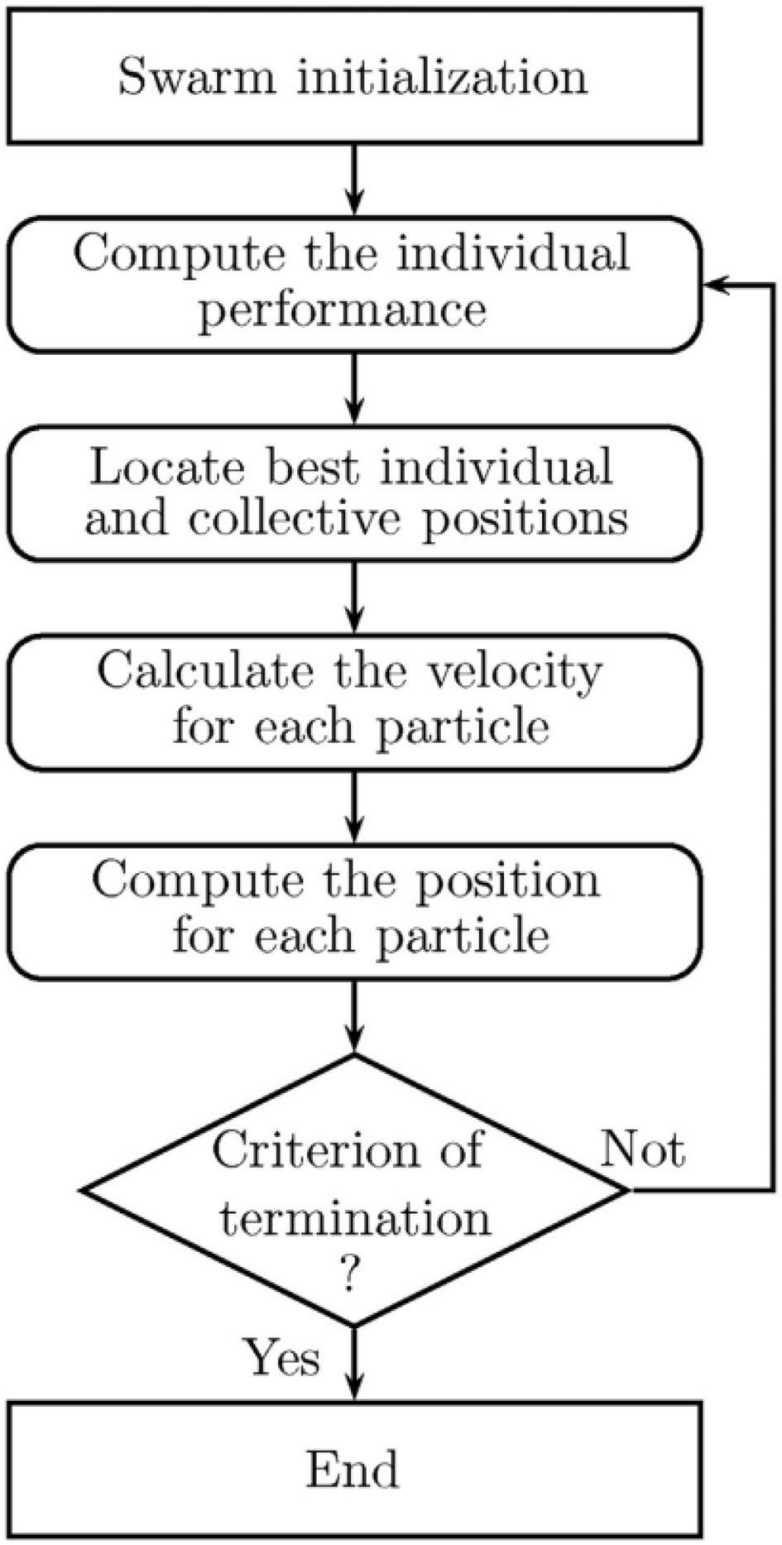
PSO algorithm^66^. In the PSO algorithm, particles move from their place in the multidimensional search spaces based on their specific speed and information over time and updates its direction to the best location (optimal solution).

PSO method discovered by observing the behavior of a group of fish and birds^64^. PSO is an evolutionary executive random search method that consists of evolutionary planning and GA and results in an optimal solution. In the PSO algorithm, each element is called a particle. These particles exist in the n-dimensional search space and move from their place in the multidimensional search spaces based on their specific speed and information over time. Each particle has enough information and updates its direction to the best place (Pbest), according to its ability. The best location is equivalent to moving the neighboring particle called Gbest. They update their particles according to the best place (Fig. 12).

#### Prediction of hybrid grain yield based on ANN

**Figure 13:**
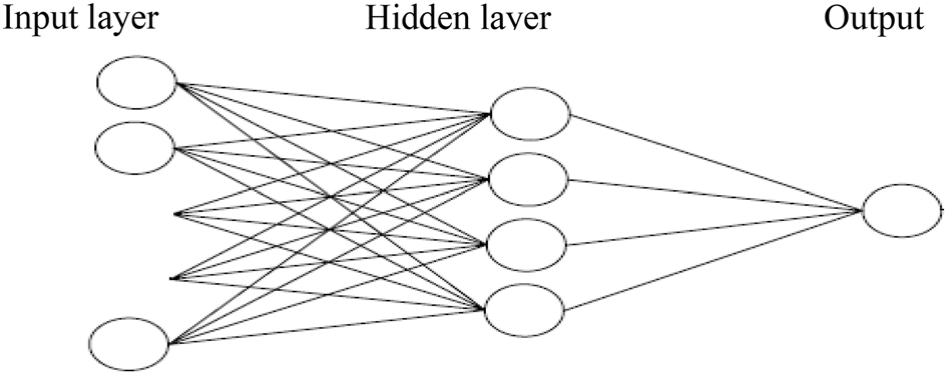
Structure of MLP. A MLP consists of input, hidden and output layers each has different neurons connected to each other.

**Figure 14:**
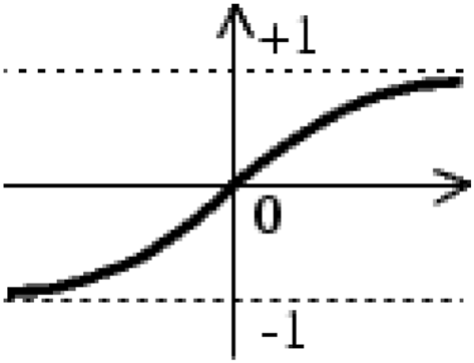
Sigmoid transfer function.

**Figure 15:**
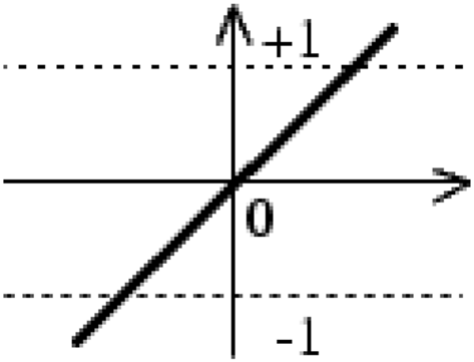
Linear transfer function.

Prediction of hybrid yield, using the measured features of the parent features, was performed with Multi-Layer Perceptron (MLP) neural network (Fig. 13). This network has three layers of neurons. The number of neurons in the first layer is equal to the number of elements of the inputs to the network. In the output layer, one neuron was used. All inputs were simultaneously applied to the network, and the weight and threshold values were adjusted after all inputs were applied to the network. The sigmoid transfer function (1) and the linear transfer function (2) were used in the hidden layer and the output layer, respectively. Figures 14 and 15 present the diagrams of these functions.

1 $$a=\frac{2}{\left(1+\mathrm{exp}\left(-2\times n\right)\right)-1}$$

2 $$a=purelin \left(n\right)=n$$

where n is the input of the neuron and $a$ is its output.

To train the network, different back propagation training algorithms were investigated. Then, all networks were trained with theses algorithms using the MATLAB R2020b software.

The selected network has various parameters that should be considered in the application of the network. These parameters were cases such as number of network epochs and the goal. In this study, the number of epochs of the network will be 200 epochs. The goal of the network is, in fact, the amount of the Mean Squared Error (MSE) value that the training algorithm stops while reaching it. To achieve the best result, this value was considered zero. To calculate network output, data, which were unknown to the network and not provided during the network training, were used. In addition, 70% of the data were used for training, 10% of the data for validation, and 20% of the data were used for testing the neural network.

To evaluate the efficiency of the neural network in predicting product performance, MSE value was used:

3 $$MSE=\frac{\sum {({y}_{t}^{\wedge }-{y}_{t})}^{2}}{n}$$

In these relationships, ${y}_{t}, {y}_{t}^{\wedge }$ and n are the actual observation value (actual value of hybrid grain yield), the predicted value of the model (neural network), and the number of observations (the number of years in the experimental group). We named the method we estimated the hybrid performance based on the neural network as AI_HIB_ANN.

### Prediction of hybrid grain yield based on SVM

Support vector, like ANN, is a type of data based algorithm. These methods are a group of supervised learning methods that are used for classification, regression, prediction and clustering problems. The problem-solving steps in a SVM, such as an ANN algorithm, are divided into two stages: training and test (validation). This method was developed based on the theory of computational learning^67^. Unlike other methods of AI, SVM, instead of reducing computational error, puts functional risk as a function of goal and its will gain optimal value. The support vector regression model is able to take the problem to a larger space by dimensions using the kernel method. In 2D space, there is an infinite number of lines to separate data from two classes. The closest training data to the hyperplane is called the support vector. The most optimal separator plane is the plane that has the maximum distance between two classes. In other words, the expression C_2_ has its maximum value^67^. According to the basics of analytic geometry

$$C= \frac{2}{\Vert W\Vert }$$

So the maximum value of C will be obtained when || W || has the lowest value. The general equation of the optimal plane will be as follows

$${W}^{T}x+b=0$$

Some data may not be in the separated range of the class. In other words, data exceeds one class and is within another class. If we assume that this degree of violation is equal to ξ, then the optimization problem becomes to find w so that the following equation is minimized:

$$Min \frac{1}{2} \Vert W\Vert +C \sum_{i}\xi i$$

Parameter C is the penalty function and its optimal value may be obtained by test or through optimization algorithms. In cases where the data is not linearly separable, the separator plane equation for the nonlinear state is obtained by interfering with the "kernel function" which is responsible for mapping the data from the nonlinear to the linear space.

The common method of Support Vector Regression (SVR) is ε -SVR. For the training data set $X=\left\{{x}_{i},{y}_{i}\right\} , i=\mathrm{1,2},\dots ,n$ approximation is done by finding a function f(x), which should not be far from the target function g(x) more than ε (i.e. $\left|f\left(x\right)-g(x)\right|<\varepsilon$). By applying a map $\mathrm{\varnothing }: {R}^{q}\to {R}^{{q}^{\mathrm{^{\prime}}}}$, with ${q}^{\mathrm{^{\prime}}}\ge q$ to the data set, the ε-SVR is shown as:

4 $${\mathrm{min}}_{\alpha ,{\alpha }^{*}}=\frac{1}{2}\sum _{i,j=1}^{n}\left({\alpha }_{i}-{\alpha }_{i}^{*}\right)\left({\alpha }_{j}-{\alpha }_{j}^{*}\right)K\left({x}_{i},{x}_{j}\right)+\varepsilon \sum _{i=1}^{n}\left({\alpha }_{i}+{\alpha }_{i}^{*}\right)-\sum _{i=1}^{n}{y}_{i}\left({\alpha }_{i}-{\alpha }_{i}^{*}\right)$$

5 $$subject \, to \left\{\begin{array}{c}{\sum }_{i=1}^{n}\left({\alpha }_{i}+{\alpha }_{i}^{*}\right)\\ 0\le {\alpha }_{i},{\alpha }_{i}^{*}\le C\end{array}\right.$$

where C is the user tuned parameter, and K is the kernel function. The kernel function on two vectors v and z is defined as:

6 $$K\left(v,z\right)=\langle \Phi \left(v\right),\Phi \left(z\right)\rangle$$

Kernel function enables the transformation of the input space into high-dimensional feature space where it is possible to apply the linear SVR algorithm. In regression problems, Gaussian one is the most common kernel function:

7 $$K{\left(x\right)}_{\left({x}_{i},{x}_{j}\right)}={\mathrm{e}}^{-\gamma \left({x}_{i}-{x}_{i}\right).\left({x}_{i}-{x}_{i}\right)}$$

After training step, the SVR function f(x) can be evaluated as follows:

8 $${y}_{i}=f \left(x\right)=\sum_{i=1}^{l}{w}_{i}K\left({x}_{i},x\right)+b$$

where x is the input vector, K is the kernel function, l is the number of the training data samples and w_i_ = (${\alpha }_{i}-{\alpha }_{i}^{*})$ is the weight vector. The vectors x_i_ corresponding to w_45231_ are called the Support Vectors (SV). The weight is usually calculated by transferring the SVR optimization problem to the dual optimization problem that equals the constrained quadratic problem and by applying quadratic programming.

We named the hybrid performance estimation method based on the SVM model AI_HIB_ SVM.

#### Prediction of hybrid grain yield based on ANFIS

Modeling using ANFIS involves two stages of training and network testing using experimental data. Of the data, 55% were used for network training and 45% for trained networks to determine the accuracy of network prediction. Therefore, networks were tested with data other than training data. Determination of the number of rules and the type of membership function is highly important. To find the best network among the other networks, networks with the number of different rules and functions in the MATLAB 2020b software environment were created using the Fuzzy Logic toolbox. As a result, network training continued until the value of the RMSE function goal was reached, or the number of epochs exceeded 100 epochs. Since the value of the RMSE function for all networks is the same, the performance of the networks can be compared. Finally, the performance of the networks in the experiment phase was compared, and the best network was selected based on the accuracy of prediction in the test phase. We named the hybrid performance estimation method based on the ANFIS model AI_HIB_ANFIS.

In the neural network model and ANFIS model, validation group datasets were used to prevent overfitting. The main purpose of using the validation group data is to determine the ability of the model to predict the output values of the experimental data using unseen input values and to prevent overfitting. When the model is well trained during the training process, the error of the validation group should be reduced. If overfitting starts, the validation error suddenly increases, indicating overfitting. In this case, the training process is stopped. In SVM model, fivefold cross validation method was used. In k-fold cross validation, the data are randomly divided into k equal parts. Each time, k-1 segments are used to train the model while the remaining 1 part is used for evaluation of model performance. This process is repeated until each part is used exactly once as a test set. After k-fold cross-validation, each data point has one observed output and one predicted output. The predicted output is the value that is calculated when the data point is placed in the test set during cross-validation. Therefore, in our opinion, the model presented in the article can be used in the real world.

## Supplementary Information

Supplementary Information 1.

Supplementary Information 2.

Supplementary Information 3.

Supplementary Information 4.

Supplementary Information 5.

Supplementary Information 6.

## Data Availability

All data generated or analysed during this study are included in this published article and Supplementary files.

## Abbreviations

AI: Artificial intelligence
ANN: Artificial neural network
MLP: Multi-layer perceptron
ANFIS: Adaptive neuro-fuzzy inference system
SVM: Support vector machine
SVR: Support vector regression
PSO: Particle Swarm optimization
GA: Genetic algorithm
TAM: Taromahalli
KHZ: Khazar
SPD: Spidroud
GHB: Gharib
AHM: Ahlamitarum
SHP: Shahpasand
GY: Grain yield
UFP: Unfertile panicle number
HE: Plant height
DF: Days to flowering
PE: Panicle exertion
PL: Panicle length
FG: Filled grain number
PBN: Primary branches number
FLL: Flag leaf length
FLW: Flag leaf width
FLA: Flag leaf area
BI: Plant biomass
