# Supplementary material for: Predicting hybrid rice performance using AIHIB model based on artificial intelligence
Source: Sci Rep. 2022 Jun 11;12:9709. doi: 10.1038/s41598-022-13805-x (PMC9188612; doi:10.1038/s41598-022-13805-x)
Supplement: Supplementary file 6 — Supplementary Information 6. [file 41598_2022_13805_MOESM6_ESM.docx]

% This is the source code for manuscript: Predicting hybrid rice performance

% using AIHIB model based on artificial intelligence

% by: Hossein Sabouri*, Sayed Javad Sajadi

load data.mat data

% data is a 9*2 table which:

% It's rows represent 9 different rice hybrids and the last row for General Data.

% It's columns represent each hybrid inputs and target.

load ga_indx.mat ga_indx

load pso_indx.mat pso_indx

% ga_indx and pso_indx are 9*1 cells which each row corresponds to the

% selected features extracted using LBBFS based on GA and PSO algorithm.

% LBBFS is a software developed at a Laboratory of system Biology and Bioinformatics,

% Institute of Biochemistry and Biophysics, university of Tehran, Tehran, Iran.

% It is available at <https://github.com/yms3786/featureselect>

%%%%%%%%%%%%%%%%%%%%%%%%%%%%%%%%%%%%%%%%%%%%%%%%%%%%%%%%%%%%%%%%%%%%%%%%%%%%%%%%%%%%%%%%%%%%%%%%%%%%%%%%%%%%%%%%%%%%%%%%%%%%%%%%%%%%%%%%%%%%%%%%%%%%%%%%%%%%%%%%%%%%%%%%%%

% Section 1

% AI_HIB_ANN: Prediction of hybrid grain yield using artificial neural network

for i = 1 : 10

d = [data.input{i,1} data.target{i,1}];

% ANN_GAPSO_USINGLBBFS is a function for train, test and calculate performance % of ANN. It is available at the end of this code at the Section 4.

Result_ANN{i,1}.GA = ANN_GAPSO_USINGLBBFS(d,ga_indx{i,1});

Result_ANN{i,1}.PSO = ANN_GAPSO_USINGLBBFS(d,pso_indx{i,1});

End

%%%%%%%%%%%%%%%%%%%%%%%%%%%%%%%%%%%%%%%%%%%%%%%%%%%%%%%%%%%%%%%%%%%%%%%%%%%%%%%%%%%%%%%%%%%%%%%%%%%%%%%%%%%%%%%%%%%%%%%%%%%%%%%%%%%%%%%%%%%%%%%%%%%%%%%%%%%%%%%%%%%%%%%%%%

%% Section 2

% AI_HIB_ SVM: Prediction of hybrid grain yield using support vector machine

for i = 1:10

d = [data.input{i,1} data.target{i,1}];

% SVR_GAPSO_USINGLBBFS is a function for train, test and calculate performance % of SVR. It is available at the end of this code at the Section 6. [Result_SVR{i,1}.GA] = SVR_GAPSO_USINGLBBFS(d,ga_indx{i,1});

[Result_SVR{i,1}.PSO] = SVR_GAPSO_USINGLBBFS(d,pso_indx{i,1});

End

%%%%%%%%%%%%%%%%%%%%%%%%%%%%%%%%%%%%%%%%%%%%%%%%%%%%%%%%%%%%%%%%%%%%%%%%%%%%%%%%%%%%%%%%%%%%%%%%%%%%%%%%%%%%%%%%%%%%%%%%%%%%%%%%%%%%%%%%%%%%%%%%%%%%%%%%%%%%%%%%%%%%%%%%%%

%% Section 3

% AI_HIB_ ANFIS: Prediction of hybrid grain yield using neuro-fuzzy inference system

for i = 1:10

d = [data.input{i,1} data.target{i,1}];

% ANFIS_GAPSO_USINGLBBFS is a function for train, test and calculate

% performance of ANFIS. It is available at the Section 7. Result_ANFIS{i,1}.GA = ANFIS_GAPSO_USINGLBBFS( d,ga_indx{i,1} );

Result_ANFIS{i,1}.PSO = ANFIS_GAPSO_USINGLBBFS( d,pso_indx{i,1} );

End

%%%%%%%%%%%%%%%%%%%%%%%%%%%%%%%%%%%%%%%%%%%%%%%%%%%%%%%%%%%%%%%%%%%%%%%%%%%%%%%%%%%%%%%%%%%%%%%%%%%%%%%%%%%%%%%%%%%%%%%%%%%%%%%%%%%%%%%%%%%%%%%%%%%%%%%%%%%%%%%%%%%%%%%%%%

%% Section 4

function result = ANN_GAPSO_USINGLBBFS(data,indx)

% This function is used to train and evaluate neural network for Prediction of hybrid

% grain yield.

%

% data : is a n*m matrix.

% n represents data samples (observations).

% m represents data features. m-1 first features are ANN inputs and the

% last column is ANN target.

% indx : selected features extracted from data based on GA and PSO algorithm.

%

% Loop for train and test ANN with different number of neurons in hidden layer and

% randomly chosen initial values.

% hn is number of neurons in hidden layer of ANN.

% each ANN structure is trained 100 times with randomly chosen initial values.

for hn = 3:50

for j = 1:100

% ANNReg is a function for train and test ANN for regression. It is

% available at the end of this code at the Section 5.

% net is the trained network.

% tr is the performance of each trained network.

% MSE is the performance of trained network expressed as Mean Squared

% Error.

[net{hn,j},tr{hn,j},MSE{hn,j}] = ANNReg(data(:,indx),data(:,end),hn);

% each network MSE of network at test phase is stored at testPerf.

testPerf(hn,j) = MSE.testPerformance;

end

end

% I_rep and I_ are indexes of minimum value of testPerf.

[temp,I_rep] = min(testPerf,[],2);

[~,I_hn] = min(temp(3:end));

% selected tr, net, MSE, I_hn and I_rep are stored at result.

result.tr = tr{I_hn+2,I_rep(I_hn+2)};

result.net = net{I_hn+2,I_rep(I_hn+2)};

result.MSE = [result.tr.best_perf result.tr.best_vperf result.tr.best_tperf];

result.I_hn = I_hn+2;

result.I_rep = I_rep(I_hn+2);

% Regression

% Linear regression between actual values and predicted by the neural network

% t is ANN target.

% y is ANN outpput.

% tTest is ANN target for test dataset.

% tTrain is ANN target for train dataset.

% tVal is ANN target for validation dataset.

% yTest is ANN ouput for test dataset.

% yTrain is ANN output for train dataset.

% yVal is ANN output for validation dataset.

% rTest and rsqr.Test are r-value and r2-value respectively.

% rTrain and rsqr.Train are r-value and r2-value respectively.

% rVal and rsqr.Val are r-value and r2-value respectively.

t = data(:,end)';

y = result.net(data(:,indx)');

%

tTest = t (result.tr.testInd);

yTest = y (result.tr.testInd);

%

[rTest,~] = regression(tTest,yTest);

result.rsqr.Test = rTest^2;

result.ty.TEST = [tTest' yTest'];

%

tTrain = t (result.tr.trainInd);

yTrain = y (result.tr.trainInd);

%

[rTrain,~] = regression(tTrain,yTrain);

result.rsqr.Train = rTrain^2;

result.ty.Train = [tTrain' yTrain'];

%

tVal = t (tr.valInd);

yVal = y (tr.valInd);

%

[rVal,~] = regression(tVal,yVal);

result.rsqr.Val = rVal^2;

result.ty.Val = [tVal' yVal'];

end

%%%%%%%%%%%%%%%%%%%%%%%%%%%%%%%%%%%%%%%%%%%%%%%%%%%%%%%%%%%%%%%%%%%%%%%%%%%%%%%%%%%%%

% Section 5

function [net,tr,MSE] = ANNReg(input,target,hiddenLayerSize)

% This function is used to train and test neural

% input is ANN inputs.

% target is ANN target.

% hiddenLayerSize is number of neurons in hidden layer of ANN.

% net is the trained network.

% tr is the performance of each trained network.

% MSE is the performance of trained network expressed as Mean Squared Error.

%

% set the options for ANN structure and training.

x = input';

t = target';

trainFcn = 'trainlm'; % Levenberg-Marquardt backpropagation learning algorithm.

net = fitnet(hiddenLayerSize,trainFcn);

net.input.processFcns = {'removeconstantrows','mapminmax'};

net.output.processFcns = {'removeconstantrows','mapminmax'};

net.divideFcn = 'dividerand'; % Divide data randomly

net.divideMode = 'sample'; % Divide up every sample

net.divideParam.trainRatio = 55/100;

net.divideParam.valRatio = 10/100;

net.divideParam.testRatio = 35/100;

net.performFcn = 'mse'; % Mean Squared Error

net.trainParam.showWindow = false;

% Train the Network

[net,tr] = train(net,x,t);

% Test the Network

y = net(x);

e = gsubtract(t,y);

performance = perform(net,t,y);

% Recalculate Training, Validation and Test Performance

trainTargets = t .* tr.trainMask{1};

valTargets = t .* tr.valMask{1};

testTargets = t .* tr.testMask{1};

MSE.trainPerformance = perform(net,trainTargets,y);

MSE.valPerformance = perform(net,valTargets,y);

MSE.testPerformance = perform(net,testTargets,y);

end

%%%%%%%%%%%%%%%%%%%%%%%%%%%%%%%%%%%%%%%%%%%%%%%%%%%%%%%%%%%%%%%%%%%%%%%%%%%%%%%%%%%%%

% Section 6

function result = SVR_GAPSO_USINGLBBFS(d,indx)

% This function is used to train and evaluate SVR for Prediction of hybrid grain

% yield.

% d : is a n*m matrix.

% n represents data samples (observations).

% m represents data features. m-1 first features are SVR inputs and the

% last column is SVR target.

% indx : selected features extracted from data based on GA and PSO algorithm.

%

% set the options for SVR structure and training.

tbl = array2table([d(:,indx{n,1}) d(:,end)]);

tbl.Properties.VariableNames{1,end}='child';

[N,~] = size(tbl);

rng default; % For reproducibility

cvp = cvpartition(N,'Holdout',0.3);

idxTrn = training(cvp); % Training set indices

idxTest = test(cvp); % Test set indices

% Train the SVR model

Mdl = fitrsvm(tbl(idxTrn,:),...

'child',...

'OptimizeHyperparameters',...

'auto',...

'HyperparameterOptimizationOptions',...

struct('AcquisitionFunctionName',...

'expected-improvement-plus'));

result.table = tbl;

result.cvp = cvp;

result.idxTrn = idxTrn;

result.idxTest = idxTest;

result.Mdl = Mdl;

% Regression

YFit_tst = predict(Mdl,tbl(idxTest,:));

[n_test,~]=size(YFit_tst);

YReal_tst=tbl.child(idxTest);

mse_test=(sum((YFit_tst-YReal_tst).^2))/n_test;

% Predict responses for the train set.

YFit_trn = predict(Mdl,tbl(idxTrn,:));

[n_trn,~]=size(YFit_trn);

YReal_trn=tbl.child(idxTrn);

mse_trn=(sum((YFit_trn-YReal_trn).^2))/n_trn;

% output of the function:

result.mse_trn = mse_trn;

result.mse_test = mse_test;

result.YReal_tst = YReal_tst;

result.YFit_tst = YFit_tst;

result.YReal_trn = YReal_trn;

result.YFit_trn = YFit_trn;

% R^2

[rtst,~] = regression(YReal_tst,YFit_tst,'one');

result.rsqr_tst = rtst^2;

end

%%%%%%%%%%%%%%%%%%%%%%%%%%%%%%%%%%%%%%%%%%%%%%%%%%%%%%%%%%%%%%%%%%%%%%%%%%%%%%%%%%%%%

% Section 7

function result = ANFIS_GAPSO_USINGLBBFS( d,indx )

% This function is used to train and evaluate ANFIS for Prediction of hybrid

% grain yield.

% d : is a n*m matrix.

% n represents data samples (observations).

% m represents data features. m-1 first features are ANFIS inputs and the

% last column is ANFIS target.

% indx : selected features extracted from data based on GA and PSO algorithm.

%

% Set the options for ANFIS

data = [d(:,indx) d(:,end)];

trn_data = data(1:2:end,:);

chk_data = data(2:2:end,:);

in_fismat = genfis3(trn_data(:,1:end-1),trn_data(:,end));

anfisOpt = anfisOptions('InitialFIS',in_fismat,...

'EpochNumber',100,...

'StepSizeDecreaseRate',0.5,...

'StepSizeIncreaseRate',1.5,...

'ValidationData',chk_data,...

'DisplayANFISInformation',0,...

'DisplayErrorValues',0,...

'DisplayStepSize',0,...

'DisplayFinalResults',0);

% Train the ANFIS

[trn_out_fismat,trn_error,step_size,chk_out_fismat,chk_error] = anfis(trn_data,anfisOpt);

[a,b] = min(chk_error);

% Test the ANFIS

anfisout_chk_data = evalfis(chk_out_fismat,chk_data(:,1:end-1));

% Result: output of this function

result.trn_data = trn_data;

result.chk_data = chk_data;

result.in_fismat = in_fismat;

result.trn_out_fismat = trn_out_fismat;

result.trn_error = trn_error;

result.step_size = step_size;

result.chk_out_fismat = chk_out_fismat;

result.chk_error = chk_error;

result.anfisout_chk_data = anfisout_chk_data;

result.RMStest = a;

result.I_RMStest = b;

result.RMStrn = trn_error(b,1);

% Regression

[rchk,~] = regression(chk_data(:,end),anfisout_chk_data,'one');

result.ty = [chk_data(:,end) anfisout_chk_data];

result.rsqrchk = rchk^2;

end
